# Impact of a sport-specific nutrition education program on knowledge and eating disorder risk in youth competitive climbers: a pilot study

**DOI:** 10.3389/fnut.2026.1767162

**Published:** 2026-02-06

**Authors:** Aaron Quesada, Ana Pablos, Eraci Drehmer

**Affiliations:** 1 Department of Physical Activity and Sports Sciences, Universidad Católica de Valencia “San Vicente Mártir”, Valencia, Spain; 2 Nutrition and Dietetics Unit, University Clinics, Universidad Católica de Valencia “San Vicente Mártir”, Valencia, Spain; 3 Departament of Health Sciences, Universidad Católica de Valencia “San Vicent Mártir”, Torrente, Spain

**Keywords:** adolescents, eating disorders, nutrition education, prevention, RED-S, sport climbing

## Abstract

**Introduction:**

Sport climbing has gained popularity among youth athletes, raising concerns about eating disorders (ED) and relative energy deficiency in sport (RED-S). Although nutritional education has shown promise in preventing ED risk, its application to young climbers remains understudied. This pilot study evaluates the impact of a tailored nutrition education program, NUTYES (“NUTrición Y EScalada”: Nutrition and Climbing), on nutritional knowledge and ED risk in competitive youth climbers, providing effect size estimates to inform future definitive trials.

**Methods:**

Twenty-three young climbers (categories U-14 and U-16, representing athletes under 14 and under 16 years of age, respectively) participated in a prospective, parallel-group pilot study. Participants were allocated to either the intervention group (IG; *n* = 11) or control group (CG; *n* = 12). Anthropometric measurements (ISAK), nutritional knowledge (GeSNK), and ED risk (EAT-26) were assessed before and after an 8-week intervention consisting of seven 2-h educational sessions focused on climbing-specific nutrition needs.

**Results:**

Post-intervention, the IG demonstrated exceptionally large improvements in sport-specific nutrition knowledge (*p* = 0.013; d = 1.37; 95% CI: 0.40–2.33) and total nutrition knowledge (*p* = 0.012; d = 1.15; 95% CI: 0.22–2.09), with significant between-group differences in change scores. General nutrition knowledge showed a large effect approaching significance (*p* = 0.073; d = 0.89; 95% CI: −0.02-1.80). No significant changes in ED risk were observed (*p* = 0.227), though a moderate effect size was detected (d = 0.58; 95% CI: −0.30-1.47) with limited statistical power (44%).

**Discussion:**

Brief sport-specific nutrition education produced exceptionally large improvements in nutritional knowledge among young climbers, with effect sizes substantially exceeding typical educational interventions in adolescent populations. While eating disorder risk showed no significant changes, the moderate effect observed suggests meaningful impacts may remain undetected due to limited statistical power and low baseline risk. These findings support the feasibility and preliminary efficacy of the NUTYES program, providing robust effect size estimates to inform future definitive trials requiring larger sample sizes (50–60 participants per group) to adequately assess eating disorder prevention outcomes.

## 1 Introduction

Sport climbing has experienced substantial growth in recent years, particularly following its inclusion in the Olympic Games (1), driving increased youth participation and early specialization in U14 and U16 competitive categories. Current scientific literature consistently identifies anthropometric and morphological characteristics as key determinants of climbing performance (2). Accordingly, elite climbers typically display body composition profiles optimized for a high strength-to-weight ratio. Systematic reviews highlight that climbers tend to exhibit markedly low body fat levels, a feature that reduces overall body mass and enhances relative strength (3). In addition, elite climbers present substantially higher lean muscle mass compared with non-climbing control groups, with reported values of approximately 86% in males and 80% in females, referring specifically to lean body mass as a percentage of total body mass (4). Disciplines within the sport further modulate these profiles: bouldering specialists generally show greater muscle mass than lead climbers, likely reflecting the greater reliance on explosive and maximal strength capacities (5, 6). While increased upper-body musculature is advantageous, disproportionate hypertrophy of the lower limbs may be counterproductive by elevating body mass and reducing movement economy (6). Consequently, functional and discipline-specific muscle development is considered essential for optimal climbing performance.

In parallel with these physical demands, adequate nutrition is essential to support training, recovery, and long-term athlete health (7). Climbers may be vulnerable to nutritional deficiencies due to the high physiological demands of the sport and widespread lack of formal nutrition education (7). Recent findings show adolescent athletes often fail to meet recommended energy and macronutrient requirements, particularly regarding carbohydrate intake, which is vital for both training and recovery (8). Current evidence on sport climbing athletes indicates significant nutritional inadequacies and potential risks of low energy availability (LEA), highlighting the importance of proper nutritional assessment and education in this population (9).

This nutritional knowledge gap is particularly concerning given growing evidence linking poor dietary literacy with elevated risk of disordered eating behaviors (DEB), LEA, and the broader clinical syndrome of RED-S (10). RED-S encompasses multiple physiological alterations resulting from chronic LEA, including metabolic, hormonal, immunological, and psychological dysfunctions. These risks are especially pronounced in esthetic or weight-sensitive sports such as climbing.

Subclinical ED and DEB have been observed among young climbers, with higher prevalence in females, although males are not exempt (11). Contemporary prevalence studies among young athletes suggest concerning rates of potential eating disorder risk, emphasizing the need for preventive interventions (12). Contributing factors include perfectionism, internalized weight standards, and pressure from coaches or peers. The early adolescent years represent a critical developmental period for body image formation, dietary behaviors, and psychosocial identity, underscoring the value of preventive strategies during this period (10).

Despite these concerning trends, research evaluating the effectiveness of nutrition education specifically in youth climbing populations remains limited. Nutrition education, particularly when tailored to the specific needs of young athletes, has shown promise in reducing RED-S and eating disorder incidence by increasing awareness, correcting misconceptions, and promoting healthier habits (13, 14). Recent studies also demonstrate the effectiveness of structured nutrition education programs in improving nutritional knowledge among young athletes (15). Although similar educational strategies have been successfully applied in other youth sports, their implementation and evaluation in competitive youth climbers are still scarce. Therefore, this pilot study aimed to assess the effectiveness of a sport-specific nutrition education program, NUTYES (“NUTrición Y ESCalada”: Nutrition and Climbing), in enhancing nutritional knowledge and potentially mitigating eating disorder risk in adolescent climbers.

## 2 Materials and methods

### 2.1 Study design

This prospective, longitudinal pilot study employed a parallel-group design to evaluate the effects of a sport-specific nutrition intervention in adolescent climbers. The study duration was 30 weeks, including recruitment, baseline assessments, intervention, and post-intervention assessments. Two evaluations were conducted: one-two weeks before the intervention (pre-intervention) and one-two weeks after its completion (post-intervention). The study was conducted between December 2022 and December 2023.

### 2.2 Participants

Twenty-three competitive youth climbers (12 males and 11 females), aged 11–15 years (categories U-14 and U-16), from climbing clubs in the Valencian Community (Spain), participated in the study. Following randomization, 11 participants were allocated to the intervention group and 12 to the control group. Inclusion criteria were: (1) active participation in regional and/or national climbing competitions; (2) regular training within a climbing club; (3) not following any specific diet; and (4) provision of informed consent by legal guardians and assent by participants. Exclusion criteria included recent cardiovascular events, acute illness, or physical limitations impeding training participation.

Clubs or training groups were assigned to intervention (IG) or control (CG) groups using computer-generated randomization to avoid assigning participants from the same training group to different conditions, which could lead to influence effects. Allocation was performed by an independent researcher using simple randomization sequence in Excel, with allocations sealed in sequentially numbered, opaque envelopes and opened after baseline assessments completion, ensuring allocation concealment and minimizing selection bias.

### 2.3 Intervention

The NUTYES (“NUTrición Y EScalada”: Nutrition and Climbing) program was developed based on a comprehensive needs assessment conducted prior to the intervention. This assessment identified specific knowledge gaps in the target population through preliminary surveys and consultation with coaches and sports nutrition experts. The program consists of seven 2-h workshops conducted over 8 weeks, delivered by a registered dietitian and a physical activity specialist. The curriculum was designed following evidence-based guidelines from the International Society of Sports Nutrition (ISSN) and the Spanish Society of Community Nutrition (SENC), adapted to the specific physiological demands of youth climbing and the Spanish dietary context. While the core nutrition principles are applicable internationally, some practical examples and food-based recommendations incorporated culturally relevant Spanish foods and eating patterns. Workshops were scheduled at regular intervals to allow athletes sufficient time to integrate knowledge into daily routines. Each session systematically began with review and discussion of questions or challenges encountered during implementation.

The educational content covered: (1) energy requirements and nutrient needs in youth athletes; (2) macronutrients and micronutrients: dietary functions and sources; (3) healthy eating patterns and food groups; (4) hydration strategies and the impact of dehydration on performance; (5) body composition in climbing: somatotypes and misconceptions related to weight; (6) food label interpretation and rating systems (e.g., Nutri-Score, NOVA); (7) nutrition for competition: pre-, during, and post-event eating strategies; (8) common nutritional deficiencies and the use of ergogenic aids in climbing; and (9) basic food safety and preparation techniques.

Interactive activities tailored to the age group were incorporated into each session to encourage participation and improve knowledge retention. Examples included nutrition knowledge quizzes, hands-on meal planning exercises, food label interpretation workshops, and group discussions on common nutrition myths in climbing. Minimum 80% session attendance was required for inclusion in the intervention group.

### 2.4 Outcome measures

Anthropometric assessments were carried out following ISAK protocols (16) and performed by a certified Level 3 anthropometrist. All measurements were obtained using validated and calibrated equipment to ensure accuracy.

Body mass was determined with a portable clinical scale (SECA model, capacity 150–200 kg, precision 100 g). Stature was then measured using a SECA 220 stadiometer (Hamburg, Germany; precision 0.1 cm), with participants properly aligned to the Frankfort plane.

Skinfold thickness was collected at eight sites (triceps, biceps, subscapular, suprailiac, supraspinal, abdominal, front thigh, and calf), using a Holtain mechanical caliper (Crymych, United Kingdom; precision 0.2 mm, range 0–40 mm).

Additionally, arm span and segment lengths were recorded using a segmometer (Cescorf, Porto Alegre, Brazil; precision 0.1 cm). Body perimeters (waist, hip, relaxed arm, and calf) were obtained using a flexible steel anthropometric tape (Lufkin W606ME model). Small bone diameters (humerus, bistyloid, femur, and bimalleolar) were measured with a Holtain bicondylar pachymeter (minimum precision 1 mm; range 0–140 mm).

Somatotype was determined according to the Heath-Carter method (17), which classifies body build into three components: endomorphy (relative fatness), mesomorphy (musculoskeletal robustness), and ectomorphy (linearity or slenderness). This method combines skinfolds, bone breadths, and limb girths to calculate a three-number rating, widely used in sports science to describe morphological characteristics.

Body compartments were estimated as follows: fat mass percentage was obtained using the Yuhasz equation (18), bone weight was calculated using the Rocha formula (19), and muscle weight was determined using the Matiegka formula (20), which then served to compute muscle mass percentage. Residual weight was estimated using the Würch formula (21). Body mass index (BMI) was obtained as body mass in kilograms divided by stature squared in meters (kg/m^2^), following Quetelet’s definition (22). Maturity offset (age at peak height velocity, APHV) was computed using the Mirwald method (23), which predicts biological maturation from anthropometric variables such as chronological age, stature, sitting height, and leg length. This non-invasive approach provides an estimate of years from peak height velocity and is widely used in growth and development research to classify maturation status in adolescent populations.

Nutritional knowledge was evaluated using the validated Spanish version of General and Sport Nutrition Knowledge Questionnaire (GeSNK) questionnaire (24). The instrument includes 62 items with two sections: General Nutrition (29 items) and Sports Nutrition (33 items). Total scores range from 0 to 97 points, with low knowledge defined as <46 points (<33rd percentile), medium as 46–58 points (33rd-66th percentile), and high as >58 points (>66th percentile).

Eating disorder risk was examined with the validated Spanish version of the Eating Attitudes Test-26 (EAT-26), a widely used 26-item screening instrument with scores ranging from 0 to 78, where values ≥20 indicate clinical risk for eating disorders (25). This validated instrument has been previously employed in adolescent athletic populations and demonstrates adequate psychometric properties for detecting subclinical eating disorder symptoms (26).

Training history and competitive experience, including years of climbing practice, competition participation, and weekly training volume, were collected through a custom-designed demographic and sports background questionnaire developed specifically for this study. The questionnaire also captured information on climbing discipline preferences (bouldering, lead, or speed) and maximal climbing grade achieved. Following the eight-week intervention period, participant satisfaction with the program content was measured using a 5-point Likert scale (1 = very dissatisfied; 5 = very satisfied).

All questionnaires were administered in person, under supervision, at the participants’ respective sports clubs to minimize disruption to their regular training schedules. Assessments were conducted in quiet, standardized settings by trained research personnel to ensure optimal comprehension, response accuracy, and consistent conditions across both pre-intervention and post-intervention measurement occasions.

### 2.5 Sample size justification and statistical analysis

As a pilot study, primary objectives were to assess feasibility, acceptability, and preliminary efficacy of the NUTYES intervention, and estimate effect sizes for future definitive trials. Sample size was determined by:

1. Population accessibility: 23 athletes met inclusion criteria and provided informed consent, representing a substantial proportion of competitive youth climbers in the area;
2. Pilot study guidelines: obtained sample size aligns with recommendations for pilot studies (12–15 participants per group) (27);
3. *Post-hoc* power analysis: study achieved ~72% power to detect large effects (d ≥ 0.8) for nutritional knowledge, based on established effect size benchmarks (28). However, *post-hoc* calculations revealed that the actual power to detect moderate effects on eating disorder risk was only 44%, limiting the ability to detect meaningful changes in this domain.

Data were analyzed using SPSS version 27 (IBM Corp., Armonk, NY, United States) and G*Power 3.1 (Universität Düsseldorf, Germany) for power analysis calculations. The Shapiro–Wilk test assessed normality of continuous variables. Descriptive statistics (mean ± standard deviation) were calculated for baseline characteristics, and independent-samples t-tests examined differences between groups at baseline. A 2 × 2 repeated-measures ANOVA assessed interaction effects of group (IG vs. CG) and time (pre- vs. post-intervention). Bonferroni-adjusted pairwise comparisons were performed for *post-hoc* analysis. Effect sizes were reported as partial eta squared (ηp^2^) from repeated measures ANOVA and as Cohen’s d for between-group differences in change scores. Partial eta squared thresholds were interpreted as small (>0.01), moderate (>0.06), or large (>0.14) according to Cohen (28). Cohen’s d was calculated as the standardized difference between groups in change scores: d = (ΔIG–ΔCG) / SD_diff, where ΔIG and ΔCG represent the change scores for intervention and control groups respectively, and SD_diff is the standard deviation of the difference scores, according to Cohen (28). Thresholds were interpreted as small (d > 0.2), moderate (d > 0.5), or large (d > 0.8). Statistical significance was set at *p* < 0.05.

Given the pilot study nature, effect size interpretation was emphasized alongside significance testing to provide comprehensive information for future study design.

## 3 Results

### 3.1 Participant flow and baseline characteristics

**Figure 1:**
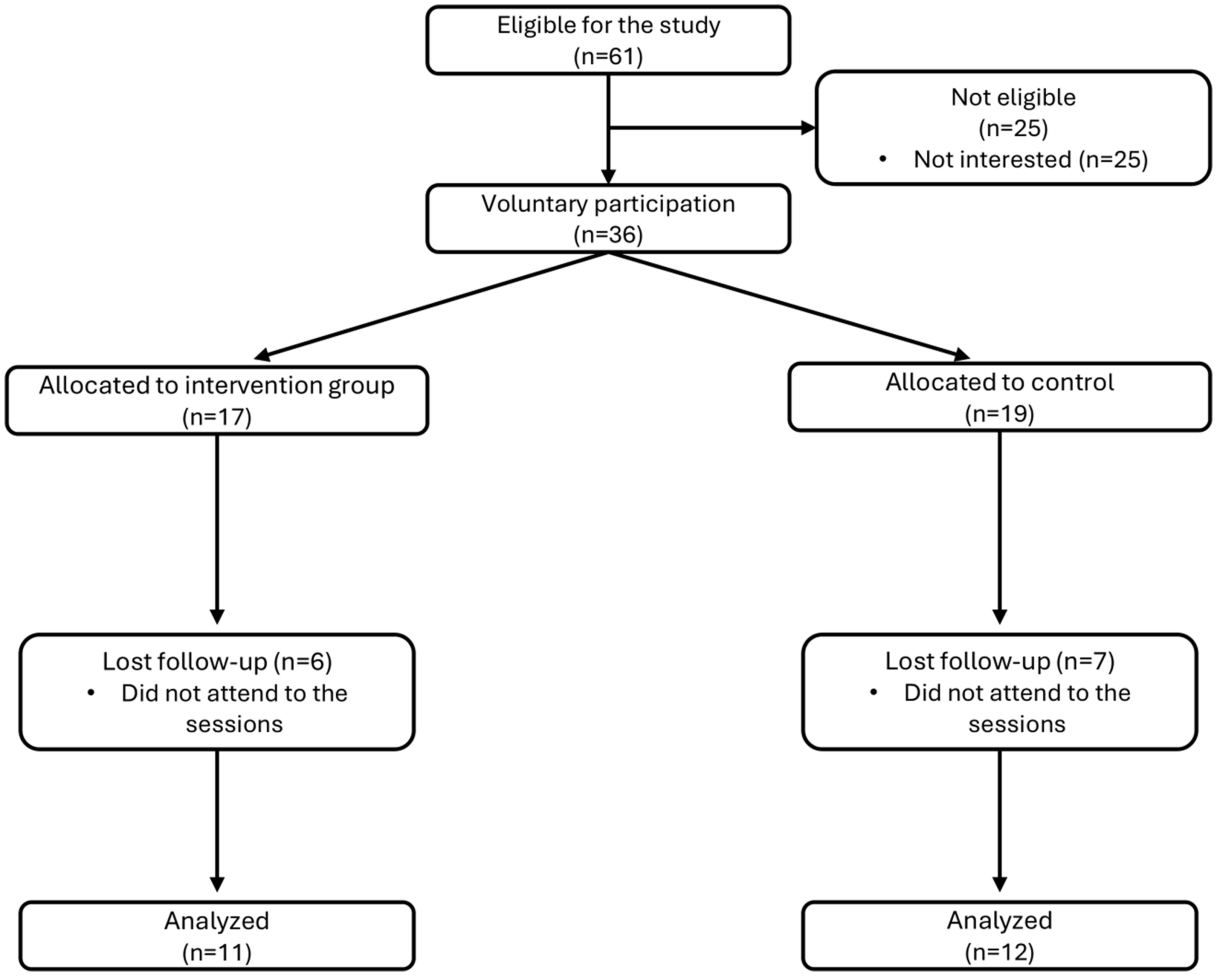
Flow chart of participant recruitment, group allocation, and completion of pre- and post-intervention assessments.

Of the 36 youth climbers initially recruited, 23 (63.9%) completed both pre- and post-intervention assessments and were included in final analysis. Participants were randomly allocated to intervention group (IG; *n* = 11), who received the nutrition education program, or control group (CG; *n* = 12), who continued regular training without specific nutritional guidance (Figure 1).

All participants met the inclusion criteria, and no adverse events were reported during the intervention period. No dropouts were related to study procedures. The demographic composition was balanced across sex (56.5% male, 43.5% female) and competitive categories (52.2% U14 and 47.8% U16).

Baseline characteristics of the study sample are presented in Table 1, including anthropometric measurements, somatotype components, and climbing-specific variables, stratified by intervention and control groups. With the exception of muscle mass percentage (IG: 45.71% ± 1.87%; CG: 47.49% ± 1.88%; *p* = 0.042), no significant differences were observed between groups, demonstrating overall group homogeneity despite the small sample size.

**Table 1:** Baseline characteristics of the study sample.

Variable	Total mean (SD)	Control mean (SD)	Intervention mean (SD)	*p*-value
Age (years)	13.02 (1.24)	13.20 (0.87)	12.65 (1.17)	0.355
Weight (kg)	43.12 (9.26)	44.02 (9.47)	42.34 (8.77)	0.677
Height (cm)	155.85 (10.67)	154.06 (9.99)	154.98 (10.93)	0.843
Seated Height (cm)	80.35 (6.26)	79.32 (6.17)	80.65 (6.06)	0.625
Arm Span (cm)	151.12 (32.42)	155.66 (9.41)	143.12 (48.82)	0.435
Sum of 6 Skinfolds (mm)	55.98 (21.04)	53.76 (15.42)	62.25 (25.72)	0.376
Sum of 8 Skinfolds (mm)	70.74 (27.91)	67.90 (20.69)	78.60 (34.42)	0.405
Body Fat (%)	10.52 (3.82)	10.42 (3.41)	11.25 (4.29)	0.630
Muscle Mass (%)	46.40 (1.99)	47.49 (1.88)	45.71 (1.87)	0.042*
Bone Mass (%)	20.10 (2.45)	19.28 (1.73)	20.11 (2.22)	0.356
Residual Mass (%)	22.99 (1.55)	22.82 (1.65)	22.94 (1.61)	0.872
BMI (kg/m^2^)	17.56 (2.20)	18.29 (1.96)	17.49 (2.23)	0.396
APHV (years)	−0.42 (1.61)	−0.34 (1.54)	−0.58 (1.50)	0.719
Leg Length (cm)	60.55 (18.25)	70.74 (11.84)	57.15 (18.84)	0.062
Foot Length (cm)	22.74 (1.34)	22.38 (1.13)	22.75 (1.39)	0.520
Hand Length (cm)	16.88 (1.19)	16.59 (1.19)	16.90 (1.20)	0.560
Endomorphy	2.53 (1.03)	2.37 (0.75)	2.86 (1.27)	0.299
Mesomorph	4.08 (1.02)	4.20 (0.65)	4.37 (1.23)	0.696
Ectomorphy	4.15 (1.29)	3.55 (0.73)	4.13 (1.41)	0.252
Climbing experience (years)	5.92 (2.56)	7.70 (1.16)	5.27 (2.94)	0.071
Competition experience (years)	3.12 (2.10)	4.20 (2.30)	2.55 (1.57)	0.102
Training frequency (hours/week)	8.94 (7.66)	9.35 (8.16)	9.64 (8.91)	0.983

BMI, Body Mass Index; APHV, Age at Peak Height Velocity; Statistical significance: *p < 0.05.

### 3.2 Nutritional knowledge and ED risk

At baseline, both intervention and control groups demonstrated baseline GeSNK scores ranging from 33.64 to 36.17 points for total nutrition knowledge (scale range: 0–97 points), indicating low level of knowledge according to established cutoffs (<46 points), with potential for improvement through targeted education (see Table 2 for detailed baseline values). Regarding eating disorder risk, baseline EAT-26 scores were consistently low in both groups (5.30–6.71 points), which is substantially below the clinical threshold of ≥20 points, indicating minimal eating disorder risk in this youth climbing population.

**Table 2:** Pre and post intervention values and time x group comparative analysis of the study variables.

Variable	Group	Pre (Mean ± SD)	Post (Mean ± SD)	F	*p*-value	ηp^2^	d	95% CI
Sport nutrition	IG	10.45 (4.13)	20.18 (5.93)	7.403	0.013*	0.261	1.37	0.40–2.33
	CG	11.33 (5.71)	14.25 (3.93)					
General nutrition	IG	23.18 (6.98)	38.54 (12.82)	3.574	0.073	0.145	0.89	−0.02-1.80
	CG	24.83 (13.08)	30.08 (9.86)					
Total nutrition	IG	33.64 (9.40)	58.73 (16.82)	7.513	0.012*	0.264	1.15	0.22–2.09
	CG	36.17 (14.10)	44.33 (12.39)					
Eating disorder risk	IG	6.71 (5.59)	10.86 (9.97)	1.584	0.227	0.096	0.58	−0.30-1.47
	CG	5.30 (2.87)	5.20 (3.39)					

The descriptive analysis values are shown as the mean (standard deviation). IG, Intervention Group; CG, Control Group. Effect sizes: ηp^2^, partial eta squared from ANOVA; d, Cohen’s d for between-group difference in change scores. Statistical significance: *p < 0.05.

A repeated measures ANOVA (Table 2) revealed significant group-time interactions for sports nutrition knowledge (*p* = 0.013) and total nutrition knowledge (*p* = 0.012), with large effect sizes indicating substantial improvements in the intervention group. General nutrition knowledge showed a non-significant trend toward improvement (*p* = 0.073), while no significant changes were observed in eating disorder risk scores (*p* = 0.227).

The intervention demonstrated large effects on sport nutrition knowledge (d = 1.37; 95% CI: 0.40–2.33) and total nutrition knowledge (d = 1.15; 95% CI: 0.22–2.09), representing clinically meaningful improvements. For general nutrition knowledge, despite the non-significant result, a large effect size was observed (d = 0.89; 95% CI: −0.02–1.80) suggesting potential clinical relevance limited by statistical power. Similarly, a moderate effect was observed for eating disorder risk (d = 0.58; 95% CI: −0.30–1.47), though this did not reach statistical significance, consistent with low baseline risk in this population.

**Figure 2:**
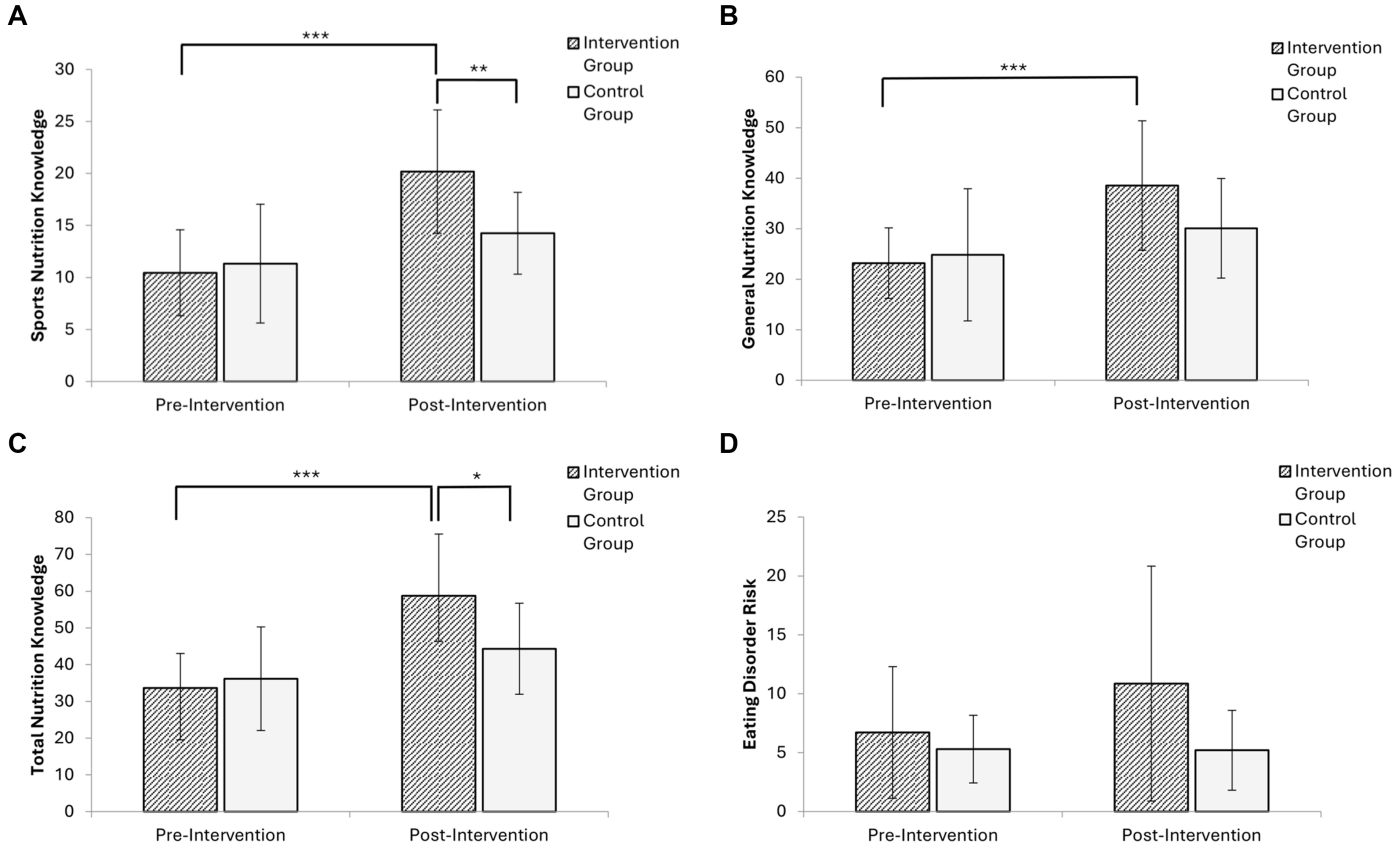
Comparison of study outcomes between groups at baseline and post-intervention: **(A)** Sport nutrition knowledge; **(B)** General nutrition knowledge; **(C)** Total nutrition knowledge; **(D)** Eating disorder risk. Statistical significance: *p < 0.05, **p < 0.01, ***p < 0.001. IG, Intervention group; CG, Control group.

*Post hoc* analyses with Bonferroni correction (Figure 2) confirmed significant within-group improvements in the intervention group across all knowledge domains, with the most pronounced gains in sport nutrition knowledge (Δ = 9.73 points; 95% CI: 6.19–13.27) and total nutrition knowledge (Δ = 25.09 points; 95% CI: 15.28–34.90). Between-group differences post-intervention were statistically significant for both sports nutrition and total nutrition knowledge.

The control group showed a modest increase in total nutrition knowledge (Δ = 8.16 points), though the magnitude was markedly lower than in the intervention group (25.09 vs. 8.16 points). For eating disorder risk, the intervention group showed an increase (Δ = 4.15 points; 95% CI: −1.66-9.96) while the control group remained virtually unchanged (Δ = −0.10 points; 95% CI: −2.11-1.91), though between-group differences were not statistically significant.

### 3.3 Adherence and feasibility

Intervention adherence was moderate, with 64.7% of participants (11/17) completing 80% of the seven educational sessions (mean attendance: 6.3 ± 0.8 sessions). Participant satisfaction was high (mean satisfaction score: 4.7/5, range: 1–5). These findings support the feasibility and acceptability of the NUTYES intervention in this population.

### 3.4 Effect size estimation for future studies

The observed effect sizes provide valuable estimates for future definitive trials. For sports nutrition knowledge, the large effect (d = 1.37) suggests that future studies could detect meaningful differences with relatively small sample sizes (8–10 participants per group for 80% power). For general nutrition knowledge, the large effect (d = 0.89) indicates that future studies would require approximately 21 participants per group to achieve 80% power. The moderate effect observed for eating disorder risk (d = 0.58) suggests that this outcome may require larger samples (approximately 50–60 participants per group) or longer follow-up periods to detect meaningful changes in low-risk populations.

## 4 Discussion

This pilot study evaluated the efficacy of the NUTYES program in improving nutritional knowledge and reducing ED risk among young competitive climbers. The intervention group demonstrated substantial improvements in both sport-specific and general nutritional knowledge, with effect sizes (d = 1.37 for sports nutrition, d = 1.15 for total nutrition) substantially exceeding typical benchmarks for educational interventions in adolescent populations. No significant changes in ED risk scores were observed, consistent with limited statistical power (44%) for detecting moderate effects in this domain.

These findings highlight the potential of brief, sport-tailored educational interventions to enhance nutrition knowledge in adolescent climbers. Improvements observed in sports nutrition knowledge align with previous studies demonstrating that targeted programs can increase awareness of energy demands, macronutrient distribution, and hydration strategies in youth sport populations (13, 15) Contemporary research further confirms that adolescent athletes often exhibit gaps in general and sport-specific nutrition knowledge, supporting the need for structured educational interventions (26). In our sample, athletes showed low baseline knowledge (GeSNK scores 33.64–36.17 points, below the cutoff of 46 points), particularly in sport-related domains, underscoring a critical need for structured nutrition education in the climbing community.

The magnitude of improvements observed in this study is particularly noteworthy. The large effect sizes for sports nutrition knowledge (d = 1.37) and total nutrition knowledge (d = 1.15) represent gains that far exceed Cohen’s conventional thresholds for large effects (d ≥ 0.8). Educational interventions in adolescent populations typically achieve small to moderate effects (d = 0.2–0.6), making these results exceptionally promising. The observed increase of 9.73 points in sport nutrition knowledge more than doubles baseline values, indicating that the NUTYES program successfully addressed a critical knowledge gap in this population.

Beyond knowledge gains, prior studies demonstrate that improvements in nutritional literacy can translate into better dietary practices and potentially reduce risk of nutritional deficiencies (15). Structured nutrition education has also been shown to positively influence energy availability awareness and eating attitudes, particularly among female athletes (29), though male populations may require tailored approaches. From a theoretical perspective, greater nutrition knowledge has been associated with healthier dietary behaviors. For example, adequate carbohydrate intake—a key nutrient for climbing performance—and certain micronutrients are often insufficient among climbers (6, 12, 14, 30). Higher nutrition knowledge has also been linked to a reduced prevalence of LEA, particularly in female athletes (31). These findings suggest that improved knowledge may serve as a protective factor against RED-S, reinforcing education as a foundational strategy in preventive health for adolescent athletes in weight-sensitive sports.

The success of the NUTYES program indicated that brief, age-appropriate, and interactive interventions implemented within a training setting can be both feasible and effective. The game-based format and continuous engagement with instructors nicely contributed to the positive outcomes. Moreover, these results emphasize the value of sport-specific content tailored to the physiological and cultural context of climbing. Given the early specialization seen in the sport and increasing concerns regarding body image and dietary control, such programs may be essential to establishing healthy nutrition habits before maladaptive behaviors develop.

Finally, the question of whether sport-specific or general nutrition knowledge should be prioritized in adolescent athletes is theoretically important and warrants careful consideration. In early adolescence (11–15 years), nutrition education typically aims primarily at establishing foundational healthy eating habits rather than highly specialized sport-specific recommendations (24). However, our findings suggest that adolescent climbers demonstrated the greatest improvement and interest in sport-specific content, possibly due to its direct perceived relevance to their athletic performance goals. The particularly large effect size observed for sport nutrition knowledge (d = 1.37) compared to general nutrition knowledge (d = 0.89) may indicate that age-appropriate, sport-tailored content serves as an engaging vehicle for broader nutrition education. Future studies might consider a progressive approach that begins with sport-specific content as a motivational entry point, then gradually introduces more comprehensive general nutrition principles to establish lasting healthy eating habits beyond the competitive years. The optimal balance between sport-specific and general nutrition education remains an important area for future investigation.

Beyond content considerations, methodological factors also warrant attention. The modest improvement observed in the control group (Δ = 8.16 points in total nutrition knowledge) raises important considerations for future research. Several factors may have contributed to this finding: (1) the general increased awareness of nutrition topics due to participation in the study, which may have prompted athletes to seek information through other channels; (2) natural maturation and ongoing learning through other sources during the intervention period; or (3) the well-documented ‘Hawthorne effect,’ whereby participants modify their behavior simply because they are being observed. Given that intervention and control participants were from different clubs and trained on different days, direct contamination between groups was unlikely. However, the study’s presence at training facilities may have heightened general nutrition awareness across all participants. This finding underscores the importance of using objective dietary intake measures alongside knowledge assessments in future trials to better distinguish between knowledge gains and actual behavioral changes.

### 4.1 Anthropometric context

The anthropometric assessment provided complementary insight into the physical profiles of participants. These profiles observed in our participants agree with previous research on young climbers, especially regarding body fat percentage and BMI. Recent research on elite lead climbers and boulderers confirms the distinctive anthropometric characteristics that optimize climbing performance, including low body fat percentages and specific muscle mass distributions (32). Nutritional assessment studies in sport climbing athletes have highlighted the importance of maintaining appropriate body composition while ensuring adequate energy availability to support both performance and health (9).

Previous research has documented body fat values in adolescent climbers that are highly consistent with those observed in our sample. Elite youth sport climbers exhibit comparable body composition characteristics, with males presenting approximately 7.23% ± 3.07% body fat and females approximately 12.79% ± 5.22% (33). Similarly, BMI values in our cohort fall within the lower range typically reported in climbing-specific studies, reflecting the critical role of an optimal strength-to-weight ratio in climbing performance (3). Overall, the anthropometric profiles identified in our athletes align with recent evidence on the morphology of elite climbers, particularly regarding the interplay between muscular development and body mass optimization (5).

Male participants in our study presented average Age at Peak Height Velocity within the expected range for their age group, indicating biological maturation according to the norm (23). Therefore, low BMI observed in this subgroup does not seem related to maturational delay but more likely to physical characteristics associated with the typical climbing body profile. Somatotype analysis revealed a mesomorphic-ectomorphic tendency in males and a more endomorphic profile in females, patterns consistent with those described in adult climber populations (4, 14, 32). These characteristics not only influence physical performance but also have potential implications for nutritional needs and susceptibility to body image-related pressures.

### 4.2 Eating disorder risk findings

Despite improvements in nutrition knowledge, no significant changes in eating disorder risk scores were detected, though this finding should be interpreted within the context of limited statistical power. Post-hoc power analysis revealed only 44% power to detect moderate effects on eating attitudes, suggesting that meaningful improvements may have occurred but remained undetected. Several factors may explain the limited observed effects.

First, the baseline risk of disordered eating in our sample was already very low (EAT-26 scores: 5.30–6.71 points out of a possible 78 points, substantially below the clinical threshold of ≥20 points), creating a pronounced “floor effect” that limits potential for measurable improvement. Contemporary research on eating disorder symptoms in adolescent athletes suggests that knowledge gaps regarding energy deficiency may contribute to elevated risk, particularly in esthetic sports (26). Second, the relatively young age of participants (11–15 years) may precede the typical emergence of eating disorder symptoms. Third, the intervention’s exclusive focus on nutrition education, without psychological or emotional health components, may have been insufficient to influence eating attitudes in the short term.

Emerging evidence suggests that multicomponent interventions, including psychological skills training, coach and parental involvement, and longer-term follow-up, are more effective in reducing ED risk among adolescent athletes (11). Recent prevalence studies among young athletes confirm concerning rates of potential eating disorder risk, highlighting the importance of comprehensive preventive approaches (12). Our findings reinforce this view, highlighting the need to broaden preventive strategies’ scope.

Indirect improvements in the control group suggest that educational messages can also be conveyed informally through peers or coaches, pointing to the importance of a supportive social environment. Extending education to families and technical staff can amplify program impact and ensure message consistency.

### 4.3 Future research directions

Extending nutrition education to families could amplify program impact and ensure message consistency, as parents and guardians are typically the primary food providers for adolescents and gatekeepers of the household food environment. Future iterations of the NUTYES program might benefit from incorporating parent education components, family-based sessions, or take-home educational materials to address the food environment beyond individual knowledge. Given the early adolescent age group studied, the involvement of families may be particularly important for translating knowledge into actual dietary behavior change.

Research among adult climbers often identifies the internet and social media as primary sources of nutrition information, though reliability concerns are well-documented regarding the quality of online nutrition content. While our study did not formally assess information source preferences or preferences for different delivery modes, the high engagement observed with interactive, in-person workshops suggests that adolescent climbers may respond well to direct, coach-facilitated education that incorporates active participation. Future studies should systematically assess preferred information sources, delivery modalities, and engagement patterns to optimize program design and potentially develop complementary digital resources as supplements to in-person education. Understanding these preferences could inform more personalized and effective nutrition education approaches for youth athletes.

### 4.4 Limitations

This pilot study has several limitations that should be considered. The small sample size (*n* = 23), determined by participant availability and pilot study guidelines, provided sufficient power (>70%) to detect significant effects on nutritional knowledge but limited power (44%) to identify moderate effects on eating disorder risk. Therefore, the absence of significant results in eating attitudes should be interpreted with caution, as *post-hoc* analysis suggests these effects may have gone undetected due to limited statistical power rather than a true lack of intervention impact.

Additionally, the intervention targeted only the young athletes, while parents and guardians—typically the primary food providers for adolescents—were not directly involved in the educational program. Given the household context of food provision, parental knowledge, attitudes, and food environments likely influence actual dietary intake beyond individual nutrition knowledge, representing a potential moderating factor not captured in our assessment. Future studies should consider incorporating family-based components to address this gap.

The geographical restriction to the Valencian Community may also limit generalization to other cultural or sporting contexts. However, the specific climbing content and standardized delivery improve potential transferability to similar youth populations internationally. The intervention duration (seven sessions over 8 weeks) was probably sufficient for knowledge acquisition but may have been too short to bring about measurable changes in behavior or attitude, which normally requires longer-term, psychosocially enriched interventions.

The absence of follow-up assessment prevents evaluation of knowledge retention or delayed effects on eating behaviors. Finally, despite high adherence, reliance on self-reported measures introduces potential response bias that should be considered when interpreting results.

### 4.5 Value as a pilot study

Despite these limitations, this pilot study successfully achieved its primary objectives of demonstrating feasibility and providing robust effect size estimates for future research. The large effect sizes observed (d > 1.0) indicate that meaningful improvements in nutritional knowledge are achievable through brief, sport-specific interventions in youth climbers. The precise effect size estimates (with narrow confidence intervals) provide valuable parameters for power calculations in future definitive trials.

For instance, future studies aiming to detect similar effects in sports nutrition knowledge would require only 8–10 participants per group to achieve 80% power, while studies targeting eating disorder risk would need larger samples (50–60 participants per group) based on our power projections. This pilot provides a strong foundation for scaling up to larger, multi-site trials while demonstrating the practical feasibility of implementing structured nutrition education in climbing training group settings.

## 5 Conclusion

This pilot study demonstrates that brief sport-specific nutrition education can produce exceptionally large improvements in nutritional knowledge among young climbers. The NUTYES program achieved effect sizes (d = 1.37 for sport nutrition, d = 1.15 for total nutrition) that substantially exceed typical educational interventions.

While eating disorder risk showed no significant changes, the moderate effect size observed (d = 0.58) combined with limited statistical power (44%) suggests that meaningful effects may remain undetected in this low-risk sample. Future studies should employ larger sample sizes and longer follow-up periods to adequately assess eating disorder prevention outcomes.

These findings support the feasibility and preliminary efficacy of the NUTYES program, providing robust effect size estimates to inform the design of future definitive trials in young climbing populations (34).

## Data Availability

The raw data supporting the conclusions of this article will be made available by the authors, without undue reservation.
